# Investigating the diagnostic efficiency of a computer-aided diagnosis system for thyroid nodules in the context of Hashimoto’s thyroiditis

**DOI:** 10.3389/fonc.2022.941673

**Published:** 2023-01-05

**Authors:** Liu Gong, Ping Zhou, Jia-Le Li, Wen-Gang Liu

**Affiliations:** The Department of Ultrasound, Third Xiangya Hospital, Central South University, Changsha, China

**Keywords:** thyroid nodule, Hashimoto’s thyroiditis, ultrasound, computer-aided diagnosis, unnecessary biopsy

## Abstract

**Objectives:**

This study aims to investigate the efficacy of a computer-aided diagnosis (CAD) system in distinguishing between benign and malignant thyroid nodules in the context of Hashimoto’s thyroiditis (HT) and to evaluate the role of the CAD system in reducing unnecessary biopsies of benign lesions.

**Methods:**

We included a total of 137 nodules from 137 consecutive patients (mean age, 43.5 ± 11.8 years) who were histopathologically diagnosed with HT. The two-dimensional ultrasound images and videos of all thyroid nodules were analyzed by the CAD system and two radiologists with different experiences according to ACR TI-RADS. The diagnostic cutoff values of ACR TI-RADS were divided into two categories (TR4 and TR5), and then the sensitivity, specificity, and area under the receiver operating characteristic curve (AUC) of the CAD system and the junior and senior radiologists were compared in both cases. Moreover, ACR TI-RADS classification was revised according to the results of the CAD system, and the efficacy of recommended fine-needle aspiration (FNA) was evaluated by comparing the unnecessary biopsy rate and the malignant rate of punctured nodules.

**Results:**

The accuracy, sensitivity, specificity, PPV, and NPV of the CAD system were 0.876, 0.905, 0.830, 0.894, and 0.846, respectively. With TR4 as the cutoff value, the AUCs of the CAD system and the junior and senior radiologists were 0.867, 0.628, and 0.722, respectively, and the CAD system had the highest AUC (*P* < 0.0001). With TR5 as the cutoff value, the AUCs of the CAD system and the junior and senior radiologists were 0.867, 0.654, and 0.812, respectively, and the CAD system had a higher AUC than the junior radiologist (*P* < 0.0001) but comparable to the senior radiologist (*P* = 0.0709). With the assistance of the CAD system, the number of TR4 nodules was decreased by both junior and senior radiologists, the malignant rate of punctured nodules increased by 30% and 22%, and the unnecessary biopsies of benign lesions were both reduced by nearly half.

**Conclusions:**

The CAD system based on deep learning can improve the diagnostic performance of radiologists in identifying benign and malignant thyroid nodules in the context of Hashimoto’s thyroiditis and can play a role in FNA recommendations to reduce unnecessary biopsy rates.

## 1 Introduction

Hashimoto’s thyroiditis (HT) is the most common autoimmune thyroid disease (AITD) (1), with a higher prevalence in women than in men (2). Lymphocytic infiltration and follicular destruction are histological features of autoimmune thyroiditis (AIT), resulting in progressive atrophy and fibrosis of the thyroid tissue (1). HT constitutes a risk factor for primary thyroid lymphoma (PTL), as clonal B cells normally present in lymphomas can be found in patients with HT, and cellular changes caused by long-term chronic antigenic stimulation may evolve into malignancy (3). Although PTL is a rare disease, it usually presents with a more aggressive course and a worse prognosis. The association between HT and thyroid cancer (TC) remains controversial, and while many studies have produced conflicting results (4–6), some scholars have highlighted an immunological link between HT and PTC (7). For these reasons, it is necessary to proactively screen patients for HT and determine whether they are accompanied by suspicious thyroid nodules. HT was clinically diagnosed based on hypothyroidism, elevated thyroglobulin antibodies (TG-Ab) and thyroid peroxidase antibodies (TPO-Ab), inhomogeneous parenchyma on ultrasonography, and lymphocytic infiltration on cytology (8). However, HT remains difficult to diagnose, with only 25%–30% of patients presenting with elevated levels of thyroid-stimulating hormone (TSH) and decreased serum thyroid hormones, and not all patients with HT have elevated TG-Ab and TPO-Ab (9). Thus, in addition to blood markers, ultrasonography and fine-needle aspiration (FNA) also become essential tools to screen for nodules in the context of HT.

HT exhibits varying ultrasound characteristics at different stages of pathology, with inhomogeneous parenchyma being the most common. The characteristics of malignant nodules underlying diffused background of HT in different studies are inconsistent, with some studies suggesting that malignant nodules in HT tend to have smooth margins and varying calcifications (10) while others suggesting irregular margins and microcalcifications (11). The diversity of malignant features leads to more ambiguous diagnostic criteria and makes it more difficult for radiologists to identify the nature of nodules in a heterogeneous background. To manage thyroid nodules effectively, many national and international associations have proposed ultrasound-based diagnostic classifications. Wang et al. compared the efficacy of three thyroid risk stratification systems and found that the TI-RADS proposed by the American College of Radiology (ACR) in 2017 was most effective for thyroid nodules in HT (12). ACR TI-RADS is able to guide whether a nodule requires FNA or follow-up based on the nodule’s category and maximum diameter (13). Nodules were scored according to five categories of features, namely, composition, echogenicity, shape, margin, and focal echogenicity, and then the scores of each feature were summed to derive the corresponding category, which is TR1 to TR5. The maximum diameter thresholds for nodules requiring FNA were as follows: TR3 nodules ≥2.5 cm, TR4 nodules ≥1.5 cm, and TR5 nodules ≥1 cm. Nevertheless, the identification of malignant features in ultrasound images is closely related to the experience of the radiologist, which leads to interobserver variability and unnecessary biopsy.

In recent years, computer-aided diagnosis (CAD) has initially been used in the screening of thyroid nodules. There are two types of CAD methods. One is classical machine learning, which builds models based on the features recognized by human experts. Many studies using support vector machines support vector machine (SVM) or random forest algorithms as classifiers found that these can improve the diagnostic accuracy of inexperienced or non-professional radiologists (14, 15), but machine learning requires human experts to extract features in the region of interest (ROI) (16), making the differences in various research results. The other is the deep learning method, which does not require prior definition by human experts (16) and can automatically extract multilevel features that cannot be recognized by radiologists (17). By contrast, deep learning approaches have significant advantages in overcoming heterogeneity using automated learning procedures (18). ITS100 (Med Imaging AI, Wuxi, China), a commercial thyroid CAD software, is an auxiliary diagnosis system based on a deep convolutional neural network (DCNN). Compared with other CAD systems, this software identifies high-dimensional features through DCNN, which enables real-time localization, characterization, and boundary segmentation of lesions in complex backgrounds, thereby eliminating the interobserver’s differences. To the best of our knowledge, most CAD systems have high diagnostic performance in diagnosing thyroid nodules without HT (17), but there are few studies using CAD systems to diagnose diffuse thyroid diseases such as HT.

Therefore, the objectives of this study were to compare the performance of the CAD system and radiologists of different seniority using ACR TI-RADS in diagnosing thyroid nodules coexistent with HT and to investigate whether unnecessary biopsy could be reduced with the assistance of the CAD system.

## 2 Materials and methods

### 2.1 Patients

This retrospective study has been approved by the Ethics Committee of the Third Xiangya Hospital of Central South University. This study involved all patients with thyroid nodules who underwent ultrasound examination in our hospital from November 2020 to November 2021. All patients signed an informed consent form.

The inclusion criteria were as follows: 1) ultrasound examination showing heterogeneous echogenicity and 2) histopathological diagnosis suggestive of Hashimoto’s thyroiditis. The exclusion criteria were as follows: 1) the patient had a history of partial thyroidectomy or coarse needle aspiration biopsy prior to the examination and 2) the images obtained were not clear.

### 2.2 Image acquisition and CAD system analysis

The two-dimensional ultrasound images and videos were acquired with the Siemens ACUSON Sequoia color Doppler ultrasound diagnostic instrument with an 18L6 transducer. The patient was placed supine on an examination bed, and the radiologist performed a dynamic scan of the transverse and longitudinal sections of the thyroid and obtained the scanning video of the thyroid nodules and the images of the largest long-axis section.

**Figure 1:**
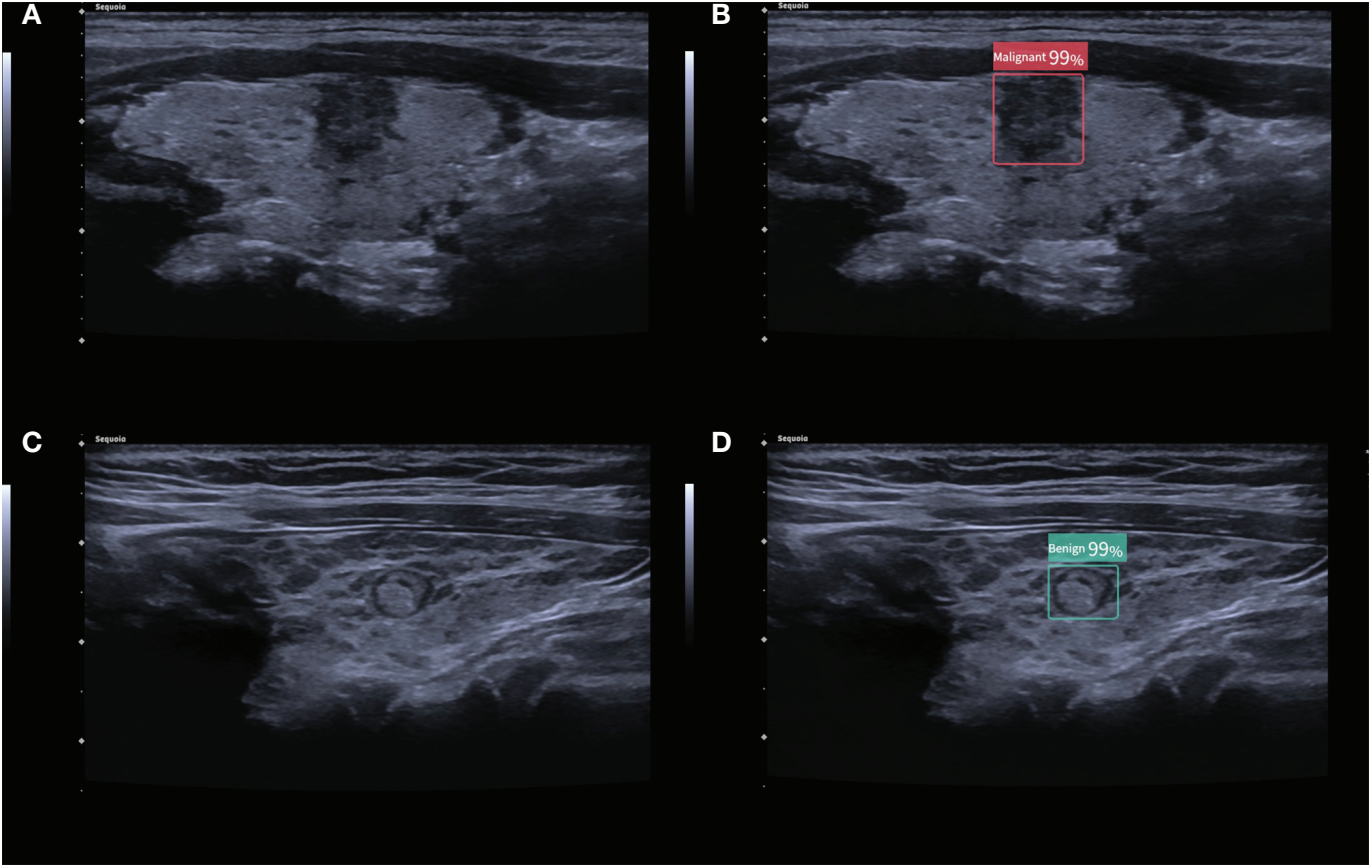
**(A)** 2D ultrasound image of a malignant thyroid nodule with HT; **(B)** the corresponding diagnostic results of the CAD system. **(C)** 2D ultrasound image of a benign thyroid nodule with HT; **(D)** the corresponding diagnostic results of the CAD system. The numbers in the figure are the benign and malignant rates produced by the CAD system. As the numbers from this CAD system are all greater than 95%, these numbers are not significant in our study.

The CAD system used in this study was Ian Thyroid Solution 100 (ITS100). The CAD system was directly connected to the ultrasonic instrument. After the collected thyroid nodule images and videos were input into the system, the location of the nodule was dynamically identified, and the malignant features in different sections were analyzed, the final results would be obtained by clicking on the screen (**Figure 1**). All of the above procedures were done by the same radiologist with over 10 years of experience.

### 2.3 Image review

The acquired 2D ultrasound images and videos were interpreted by another senior radiologist (experience over 10 years) and a junior radiologist (experience within 5 years), who analyzed the thyroid nodules according to the ACR TI-RADS (13). They were blinded to the pathological findings of the thyroid nodules and the results of the CAD system before interpretation.

### 2.4 CAD system revised recommendation of FNA

**Figure 2:**
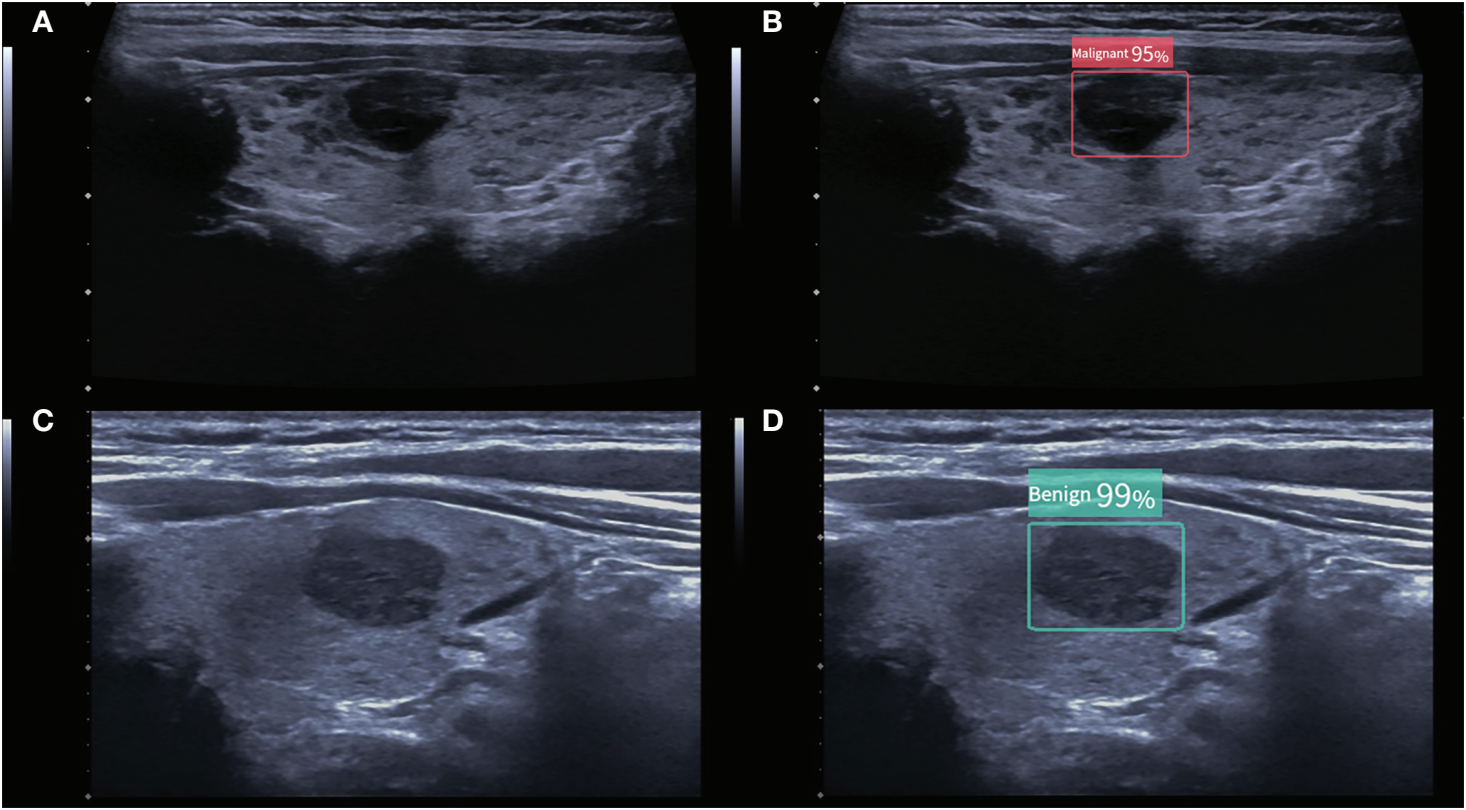
**(A)** A malignant thyroid nodule measuring 11 × 9 mm. The ACR TI-RADS of the nodule given by two radiologists were all TR4, and follow-up was recommended. **(B)** The CAD system indicated that the nodule was malignant, and the modified ACR TI-RADS was TR5, with a recommendation for FNA. **(C)** A benign thyroid nodule measuring 15 × 9 mm. The ACR TI-RADS of the nodule given by the junior radiologist was TR4, and FNA was recommended. **(D)** The CAD system indicated that the nodule was benign, and the modified ACR TI-RADS was TR3, with a recommendation for follow-up. The numbers in the figure are the benign and malignant rates produced by the CAD system. As the numbers from this CAD system are all greater than 95%, these numbers are not significant in our study.

The ACR TI-RADS provides clear guidance on whether to perform FNA or follow-up of the thyroid nodules. The criteria for using the CAD system to revise the ACR TI-RADS recommendations for FNA are as follows: when the CAD system indicated that the thyroid nodule was benign, one level was subtracted to the ACR TI-RADS except TR1. Conversely, when the CAD system indicated that the thyroid nodule was malignant, the ACR TI-RADS classification was increased by one level except TR5. According to the modified ACR TI-RADS, the distribution of benign and malignant nodules in each classification was recorded, and suggestions on whether the nodules should be FNA were provided (**Figure 2**).

### 2.5 Statistical analysis

The cutoff value of ACR TI-RADS for benign and malignant thyroid nodules was divided into two categories. The first category took TR4 as the cutoff value; TR1, TR2, and TR3 as possibly benign; and TR4 and TR5 as possibly malignant. The second category took TR5 as the cutoff value; TR1, TR2, TR3, and TR4 as possibly benign; and TR5 as possibly malignant. Using surgical pathology results as the gold standard, the receiver operating characteristic (ROC) curve of CAD and radiologists with different cutoff values was established. The sensitivity, specificity, positive predictive value (PPV), negative predictive value (NPV), accuracy, and area under the ROC curve (AUC) were calculated, respectively.

Statistical analyses were performed with SPSS 26.0 and MedCalc 19.0.4. Continuous variables were expressed as mean ± standard deviation. The age of the patients, blood markers, and the maximum diameter of the nodules were compared using *t*-test (or Mann–Whitney *U* test), and the gender of patients and the sonographic features of the nodules were compared using the chi-square test (or Fisher’s exact test). The Delong’s test was used to determine whether there was any statistical difference in diagnostic efficacy between the CAD system and the radiologists, and the McNemar test was used to compare sensitivity and specificity. *P <*0.05 indicated statistically significant differences.

## 3 Results

### 3.1 Demographics and thyroid nodule characteristics

In total, 242 consecutive patients were eligible for ultrasound suggestive of heterogeneous echogenicity and histopathological diagnosis suggestive of HT; however, 41 cases were excluded because of a history of partial thyroidectomy, 12 cases because of a coarse needle aspiration biopsy prior to examination, and 52 cases because of unclear images that prevented recognition by the CAD system. Ultimately, a total of 137 HT patients (mean age, 43.5 ± 11.8 years) were enrolled in this study, consisting of 53 benign patients and 84 malignant patients. In the benign group, some of the patients underwent surgery for suspicious pseudo-nodules. Some of these patients had indeterminate FNA results coupled with ultrasound suggesting possible malignancy, they were treated surgically for safety, and a few other patients underwent surgery for large cystic–solid mixed nodules, with final histopathological findings of pseudo-nodules in 36 cases, nodular goiters in 13 cases, and thyroid adenomas in 4 cases, respectively. In the malignant group, all cases were PTC. The basic characteristics of the patients and nodules are summarized in **Table 1**. There were no significant differences in gender (*P* = 0.457) and maximum tumor diameter (*P* = 0.118) between the benign and malignant groups. However, patients with benign nodules were younger than those with malignant nodules (*P* < 0.001), and the differences in blood markers associated with HT between the groups were not statistically significant (all *P* > 0.05). By comparing the sonographic features of benign and malignant nodules, we observed that there was no remarkable difference in margin (*P* = 0.065), but statistically significant differences in composition, echogenicity, aspect ratio, and presence of microcalcifications (all *P* < 0.05).

**Table 1:** The characteristics of patients and nodules.

Characteristics	Total	Benign nodules	Malignant nodules	*P*-value
Sex	–	–	–	0.457
Male	43	19	24	–
Female	94	34	60	–
Age	43.5 ± 11.8	48.2 ± 11.2	40.5 ± 11.2	<0.001
Tumor diameter (mm)	11.2 ± 8.3	12.8 ± 11.1	10.2 ± 5.7	0.118
Blood markers				
TSH	1.6 ± 1.2	1.4 ± 1.3	1.7 ± 1.1	0.316
TG	17 ± 17.4	19.4 ± 18.4	14.6 ± 16.4	0.349
TG-Ab	132.4 ± 263.1	123.7 ± 180.8	141.1 ± 329.6	0.822
TPO-Ab	196.7 ± 281.4	184.6 ± 272.4	208.8 ± 295.4	0.769
Composition				0.028
Solid	83	26	57	
Mixed cystic and solid	54	27	27	
Echogenicity				0
Hypoechoic	113	33	80	
Hyperechoic or isoechoic	24	20	4	
Shape				0.003
Taller than wide	52	12	40	
Wider than tall	85	41	44	
Margin				0.065
Lobulated or irregular	88	29	59	
Smooth or ill-defined	49	24	25	
Calcifications				0
Microcalcifications	78	17	61	
No microcalcifications	59	36	23	

### 3.2 Diagnostic performance of CAD and radiologists

**Figure 3:**
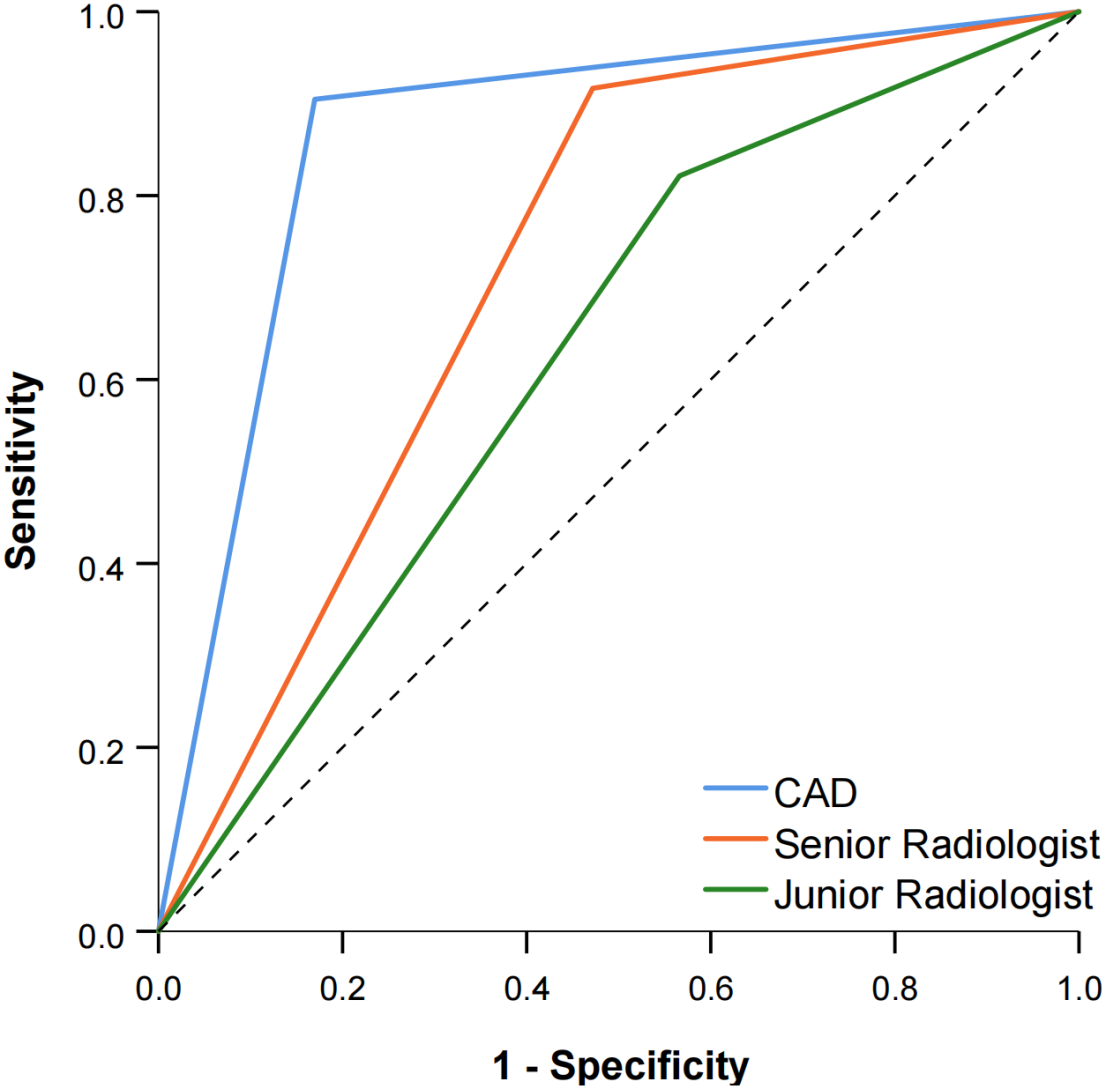
Comparison of the ROC curves between CAD and the two radiologists when TR4 was used as the cutoff value.

**Figure 4:**
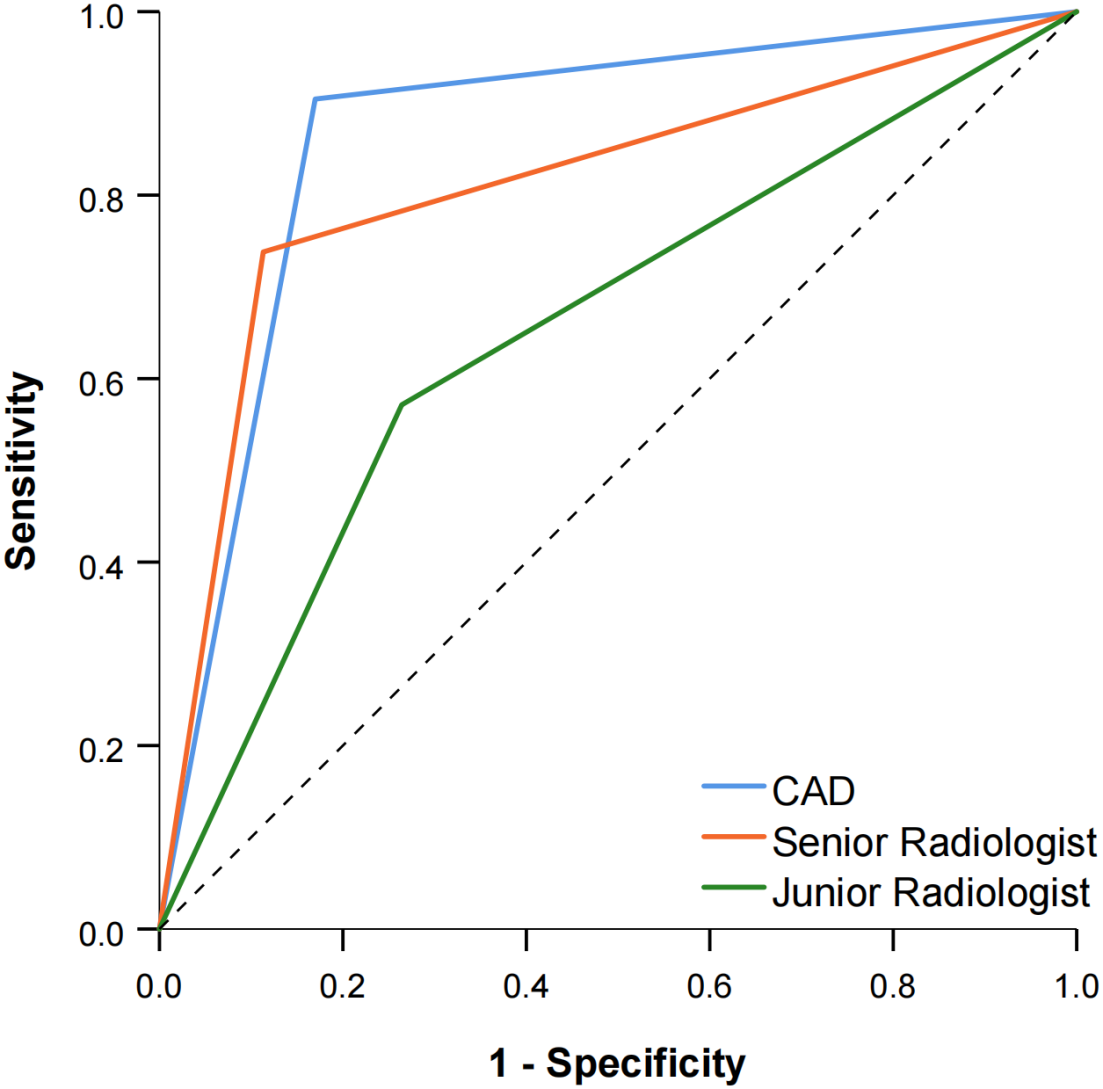
Comparison of the ROC curves between CAD and the two radiologists when TR5 was used as the cutoff value.

The diagnostic performance of the CAD software and the junior and senior radiologists for thyroid nodules with HT is shown in **Table 2** and compared in **Table 3**. When TR4 was used as the cutoff value, the AUCs of the CAD system and the junior and senior radiologists were 0.867, 0.628, and 0.722, respectively. The CAD system had the highest AUC (*P* < 0.0001), and the specificity of the CAD system was significantly higher than that of both the junior and senior radiologists (*P* < 0.0001), but there was no statistical difference in sensitivity between the CAD system and all radiologists (*P* > 0.05). When TR5 was used as the cutoff value, the AUCs of the CAD system and the junior and senior radiologists were 0.867, 0.654, and 0.812, respectively, and the AUC of the CAD system was superior to that of the junior radiologist (*P* < 0.0001) but not different from that of the senior radiologist (*P* = 0.0709). The sensitivity of CAD was higher than that of both the junior and senior radiologists (all *P* < 0.05), but the specificity was lower than that of the senior radiologist (*P* = 0.0375) and not significantly different from that of the junior radiologist (*P* = 0.1797). The ROC curves for the CAD system, senior radiologist, and junior radiologist under different cutoff values are shown in **Figures 3**, **4**.

**Table 2:** Quantitative indicators of the CAD system and the two radiologists with different seniority.

Method	AUC		Accuracy	Sensitivity	Specificity	PPV	NPV
Cutoff value TR4							
CAD	**0.867**	**0.876**		**0.905**	**0.830**	**0.894**	**0.846**
Senior radiologist	0.722	0.766		0.918	0.428	0.755	0.800
Junior radiologist	0.628	0.672		0.821	0.434	0.697	0.605
Cutoff value TR5							
CAD	**0.867**		**0.876**	**0.905**	0.830	0.894	**0.846**
Senior radiologist	0.812		0.796	0.738	**0.887**	**0.912**	0.681
Junior radiologist	0.654		0.635	0.571	0.736	0.774	0.520

The bold values represent the best value of an index in the comparative experiments.

AUC, area under the receiver operating characteristic curve; PPV, positive predictive value; NPV, negative predictive value.

**Table 3:** Quantitative indicators for the two radiologists compared with the CAD system.

	*P*-value			
	Sensitivity	Specificity	AUC	
Cutoff value TR4				
Senior radiologist	1.000	<0.0001*	<0.0001*	
Junior radiologist	0.0923	<0.0001*	<0.0001*	
Cutoff value TR5				
Senior radiologist	0.0005*	0.0375*	0.0709	
Junior radiologist	0.0012*	0.1797	<0.0001*	

*Represents statistical significance.

### 3.3 Effectiveness of the CAD system in reducing unnecessary biopsy

The modified ACR TI-RADS classification is presented in **Table 4**. Compared with the senior radiologist alone, the modified ACR TI-RADS TR3 malignancy rate was lower (20.0% vs 11.1%), and the malignancy rate for both TR4 and TR5 was higher (44.1% vs 83.3%, 91.3% vs 92.8%). Meanwhile, 13 malignant nodules raised from TR4 to TR5, four malignant nodules changed from TR3 to TR4, 15 benign nodules lowered from TR4 to TR3, and 27 benign nodules downgraded from TR3 to TR2. The diagnostic efficacy of the junior radiologist also improved with the assistance of the CAD system, the modified ACR TI-RADS TR3 malignancy rate decreased (39.5% vs. 16.7%), and TR4 and TR5 malignancy rates increased (56.8% vs. 63.2% and 77.4% vs. 88.0%). Furthermore, 18 malignant nodules upgraded from TR4 to TR5, 12 malignant nodules increased from TR3 to TR4, 14 benign nodules lowered from TR4 to TR3, and 22 benign nodules decreased from TR3 to TR2.

**Table 4:** Modified ACR TI-RADS according to the CAD system.

ACR TI-RADS	Total	Benign nodules	Malignant nodules	Malignant rate (%)
Senior radiologist/+CAD				
2	0/30	0/30	0/0	0/0
3	35/18	28/16	7/2	20.0/11.1
4	34/6	19/1	15/5	44.1/83.3
5	68/83	6/6	62/77	91.3/92.3
Junior radiologist/+CAD				
2	0/25	0/25	0/0	0/0
3	38/18	23/15	15/3	39.5/16.7
4	37/19	16/7	21/12	56.8/63.2
5	62/75	14/9	48/66	77.4/88.0

The diagnostic efficacy of the radiologists assisted by the CAD system and the comparison with the radiologist alone are shown in **Tables 5**, **6**. Using the modified ACR TI-RADS TR4 as the cutoff value, the AUCs of the senior radiologist + CAD system and the junior radiologist + CAD system were 0.876 and 0.813, respectively, both of which were higher than the performance of the radiologist alone (all *P* < 0.0001). The specificity of the combined diagnosis was also higher than that of the radiologist alone for both junior and senior radiologists (all *P* = 0.0001). When the modified ACR TI-RADS TR5 was used as the cutoff value, the AUCs of the senior radiologist + CAD system and the junior radiologist + CAD system were 0.856 and 0.808, respectively, and the diagnostic efficacy of the junior radiologist + CAD system was higher than when diagnosed alone (*P* < 0.0001), and there was no significant difference between the senior radiologist with or without the assistance of the CAD system (*P* = 0.1457). The sensitivity of all radiologists with the CAD system was higher than the diagnosis alone (all *P* < 0.05).

**Table 5:** Quantitative indicators of the two radiologists with different seniority assisted by the CAD system.

Method	AUC		Accuracy	Sensitivity	Specificity	PPV	NPV	
Cutoff value modified TR4								
Senior radiologist + CAD	0.876	0.891		0.940	0.811	0.888	0.896
Junior radiologist + CAD	0.813	0.839		0.928	0.698	0.830	0.860
Cutoff value modified TR5								
Senior radiologist + CAD	0.856		0.861	0.881	0.830	0.892	0.815
Junior radiologist + CAD	0.808		0.803	0.786	0.830	0.880	0.710

**Table 6:** Quantitative indicators of the two radiologists combined with the CAD system compared with the radiologists alone.

	*P*-value		
	Sensitivity	Specificity	AUC
Cutoff value modified TR4			
Senior radiologist + CAD	0.6875	0.0001*	<0.0001*
Junior radiologist + CAD	0.0352*	0.0001*	<0.0001*
Cutoff value modified TR5			
Senior radiologist + CAD	0.0018*	0.3750	0.1457
Junior radiologist + CAD	<0.0001*	0.1797	<0.0001*

*Represents statistical significance.

The effect of the CAD system to reduce unnecessary biopsy is demonstrated in **Table 7.** With the assistance of the CAD system, the malignancy rate of biopsy increased from 60.8% to 82.8% for senior radiologists and from 48.2% to 78.3% for junior radiologists. Compared with radiologists alone, the unnecessary biopsy rate of the senior radiologist + CAD was reduced (14.6% vs 7.3%, *P* = 0.026), and the unnecessary biopsy rate of the junior radiologist + CAD was more significantly reduced (20.4% vs 9.5%, *P* = 0.003).

**Table 7:** The effectiveness of the CAD system in assisting junior and senior radiologists in FNA.

	FNA		Malignant rate of biopsy (%)	Unnecessary biopsy rate (%)	*P*-value
	Benign	Malignant			
Senior radiologist	20	31	60.8 (31/51)	14.6 (20/137)	–
Junior radiologist	28	26	48.2 (26/54)	20.4 (28/137)	–
Senior radiologist + CAD	10	48	82.8 (48/58)	7.3 (10/137)	0.026*
Junior radiologist + CAD	13	47	78.3 (47/60)	9.5 (13/137)	0.003*

P-value is the comparison of unnecessary biopsy rates before and after the application of the CAD system.

*Represents statistical significance.

## 4 Discussion

As far as we know, several studies have shown that chronic lymphocytic thyroiditis is somehow associated with the development of both PTC and PTL (4, 7, 19), and one case of a patient with both PTC and PTL arising in the setting of HT has been reported (20), such that the treatment and prognosis of the patient would be altered. Ultrasonography and FNA assist in the early identification of patients with malignant changes and should be used promptly in uncertain situations (21). Since FNA is an invasive examination and the ultrasound characteristics of HT make it difficult to identify and aspirate the nodules (22), ultrasound is widely applied in the diagnostic of HT as a non-invasive tool. Conventional sonographic features of HT often present as diffuse parenchyma with numerous inflammatory pseudo-nodules that interfere with the radiologist’s judgment, and ultrasound relies on the experience of the radiologist. It can be challenging for young radiologists in remote areas to diagnose thyroid nodules with HT.

Recently, CAD has begun to be used in the diagnosis of thyroid nodules. The results of many studies have shown that the diagnostic efficacy of CAD systems was comparable to that of experienced radiologists and higher than that of inexperienced radiologists (23–27). Gao et al. found that CAD systems possessed a higher sensitivity than experienced radiologists when the ACR TI-RADS TR5 was used as the cutoff value (0.967 *vs*. 0.900, *P* < 0.01) (28). It should be noted that patients with HT were excluded from the above study in order to avoid interference of the CAD system by the complicated context. Hence, different CAD systems had shown high diagnostic performance for thyroid nodules in a homogeneous background. However, such CAD systems were not applicable to all people, and it is our expectation that the CAD system can effectively identify thyroid nodules in a complex background.

Feature selection and extraction are essential steps for traditional CAD systems. It mainly includes four kinds of features, namely, texture features, morphological features, model-based features, and descriptor features (29), but most of the features are artificially designed, and deep learning CAD systems can automatically extract high-dimensional features that humans cannot recognize. Therefore, some scholars have attempted to use a deep learning CAD system to diagnose thyroid nodules within a complex context. Zhao et al. developed an HT-CAD model based on the convolutional neural network (CNN) with higher diagnostic performance than senior radiologists (*P* < 0.001), and the accuracy was improved by nearly 9% (30). Hou et al. used a deep learning-based CAD system to distinguish nodules in the context of HT, and the AUC was significantly higher than that of three groups of radiologists with different years of experience (all *P* < 0.05) (31). The above studies demonstrated the diagnostic feasibility of CAD systems based on deep learning for nodules in HT, but only static images of the largest section of the nodule were input into the CAD system. As we all know, the malignant features of thyroid nodules vary in different angles and sections, and many nodules with HT have irregular margins and indistinct borders. In this scenario, the CAD system based on deep learning exploited in this study takes advantage of its ability to automatically identify and track the malignant features of each section during dynamic scanning, which can more accurately distinguish the nature of nodules and reduce the interference caused by individual sections compared with static image recognition alone.

Many studies have shown that the CAD system is more beneficial for junior radiologists to improve their diagnostic performance (32–34), which was also confirmed in our study. In this study, we used ACR TI-RADS TR4 and TR5 as the cutoff values separately and found that the AUC of the CAD system was higher than that of both junior and senior radiologists when TR4 was used as the cutoff value, and the specificity of the radiologists was lower than that of the CAD system. The AUC of the CAD system was higher than that of junior radiologists and comparable to that of senior radiologists when TR5 was used as the cutoff value, and the sensitivity of radiologists at this time is lower than that of the CAD system. The reason for this discrepancy may be due to the less distinctive malignant features of nodules in TR4 compared with TR5, resulting in less specificity for radiologists and more reliance on CAD systems. Consequently, we can conclude that no matter whether the ACR TI-RADS cutoff value of TR4 or TR5 was used, the CAD system showed higher diagnostic performance than junior radiologists and was greater than or comparable to senior radiologists. Other than that, regardless of whether the modified ACR TR4 or TR5 was used as the cutoff value, the efficacy of the junior radiologist + CAD was higher than that of the radiologist alone, while the efficacy of the senior radiologist was not significantly different from that of the radiologist alone, which could indicate that the support of the CAD system is more contributing to the diagnostic effectiveness of junior radiologists.

FNA is often performed because of suspicious nodules in patients with HT (35). It was found in our study that radiologists combined with the CAD system, compared with radiologists alone, resulted in a decreased number of TR4 nodules and an increased number of TR2 and TR5 nodules. The junior radiologist, assisted by the CAD system, moved 30 malignant nodules up one level and 43 benign nodules down one level. According to the modified ACR TI-RADS recommendations for FNA, the rate of unnecessary biopsy rate by junior and senior radiologists decreased by 10.9% and 7.3%, and the rate of malignancy in punctured nodules increased by 30.1% and 22.0%, respectively, which were consistent with the results of several studies (23, 36, 37). Therefore, with the assistance of the CAD system, radiologists were capable of reducing unnecessary biopsy of thyroid nodules in the context of HT and improving the malignancy rate of the nodules.

The present study has also some limitations. First, this study is a single-center study with a small sample size, and all patients included in this study were surgical patients, so these limitations increased the possibility of bias. The second is that all of the malignant thyroid nodules in this study were papillary carcinoma, while other pathological types, such as medullary cancer or lymphoma, need to be investigated. In addition, the background of nodules with HT is complex, which makes the automatic identification of nodules difficult, and further development of a higher-performance CAD system is required. A multicenter, prospective study exploring the value of deep learning CAD software in HT with a large sample of different types of TC is worthy of further development.

## 5 Conclusion

In conclusion, this study shows that the CAD system based on deep learning is a non-invasive and effective method to identify benign and malignant thyroid nodules in the context of HT. Moreover, radiologists, with the assistance of the CAD system, can play a role in FNA recommendations and reduce the rate of unnecessary biopsies, especially for junior radiologists.

## Data availability statement

The original contributions presented in the study are included in the article/supplementary material. Further inquiries can be directed to the corresponding author.

## Ethics statement

Written informed consent was obtained from the individual(s) for the publication of any potentially identifiable images or data included in this article.

## Author contributions

LG was responsible for the writing of the manuscript and the design of the study. PZ provided major suggestions for the manuscript. JL and WL advised on the data analysis and image collection. All authors contributed to the article and approved the submitted version.
